# The impact of signaling pathways on the desmosome ultrastructure in pemphigus

**DOI:** 10.3389/fimmu.2024.1497241

**Published:** 2025-01-15

**Authors:** Thomas Schmitt, Julia Huber, Julia Pircher, Enno Schmidt, Jens Waschke

**Affiliations:** ^1^ Chair of Vegetative Anatomy, Institute of Anatomy, Faculty of Medicine, Ludwig-Maximilan-Universität (LMU) Munich, München, Germany; ^2^ Lübeck Institute of Experimental Dermatology, University of Lübeck, Lübeck, Germany; ^3^ Department of Dermatology, University of Lübeck, Lübeck, Germany

**Keywords:** adhesion, desmosomes, pemphigus, autoantibodies, autoimmune disease, skin, epidermis

## Abstract

**Introduction:**

The autoantibody-driven disease pemphigus vulgaris (PV) impairs desmosome adhesion in the epidermis. In desmosomes, the pemphigus autoantigens desmoglein 1 (Dsg1) and Dsg3 link adjacent cells. Dsgs are clustered by plaque proteins and linked to the keratin cytoskeleton by desmoplakin (Dp). The aim of this study was to identify the impact of several PV-related signaling pathways on desmosome ultrastructure.

**Methods:**

STED microscopy, Dispase-based dissociation assay.

**Results:**

As observed using STED microscopy, pemphigus autoantibodies (PV-IgG) reduced desmosome number, decreased desmosome size, increased plaque distance and thickness and caused loss of adhesion. Decreased desmosome number, increased plaque distance and thickness and loss of adhesion correlate with features found for newly assembled immature desmosomes, observed after Ca^2+^ depletion and repletion. This was paralleled by plaque asymmetry, keratin filament retraction and fragmentation of Dsg1 and Dsg3 immunostaining. Inhibition of each individual signaling pathway investigated here prevented the loss of adhesion and ameliorated keratin retraction. In addition, inhibition of p38MAPK or PLC completely rescued all parameters of desmosomes ultrastructure and increased desmosome number under basal conditions. In contrast, inhibition of MEK1/2 was only partially protective for desmosome size and plaque thickness, whereas inhibition of Src or increase of cAMP decreased desmosome size but increased the desmosome number even in the presence of PV-IgG.

**Discussion:**

Alterations of the desmosomal plaque ultrastructure are closely related to loss of adhesion and regulated differently by signaling pathways involved in pemphigus pathogenesis. This insight may allow identification of novel treatment options targeting specific steps of desmosome turn-over in the future.

## Introduction

The blistering autoimmune skin disease pemphigus is caused by autoantibodies mainly targeting the desmosomal cadherins desmoglein (Dsg)1 and Dsg3 (1–3). The autoantibody profiles with either anti-Dsg1 or anti-Dsg1 and anti-Dsg3 IgG largely correlate with the clinical phenotypes of pemphigus foliaceus (PF) or pemphigus vulgaris (PV) respectively (1, 2). However, Dsg3 epitope-specific effects were also observed to potentially contribute to differences in clinical phenotypes (4). Autoantibodies against other targets, for example desmocollin 3, can also play a role (5–14). This demonstrates that the overall pathology is complex. Desmosomal cadherins are clustered into desmosomes by plaque proteins including plakoglobin (Pg) and anchored to the keratin cytoskeleton via the linker protein desmoplakin (Dp). Pemphigus autoantibodies can both directly inhibit cadherin interaction and activate downstream signaling pathways which interfere with desmosome assembly and remodeling (15–18). It was demonstrated before that pemphigus IgGs impact several parameters of the desmosome ultrastructure, including decreased desmosome size, increased plaque thickness and inter-plaque distance as well as reduced desmosome number (17, 19). These alterations may be representative for immature desmosomes (20), which fits to the hypothesis that pemphigus IgGs induce desmosome disassembly and cause disturbances in desmosome assembly (1, 18). It was furthermore shown that modulation of pemphigus-IgG-induced signaling can ameliorate alterations of the desmosome ultrastructure and prevent loss of adhesion (17, 19). The best described signaling molecule in this context is p38MAPK, known to play a central role in pemphigus pathogenesis. The stress kinase p38MAKP (21–23) is associated with Dsg3 and Pg (24, 25) and activates a broad spectrum of downstream targets causing both direct and transcriptional effects (18). p38MAPK was shown to be involved in depletion of Dsg3 from the cell borders (21, 26, 27) keratin filament remodeling (28–30) and loss of adhesion *in vitro* (31, 32). p38MAPK inhibition rescued PV-IgG-induced desmosome number, size, keratin dissociation and prevented desmosome splitting, ultimately preventing blister formation in human skin *ex vivo* (32, 33). Another target is PLC, inducing rapid Ca^2+^ influx and release of diacylglycerol, activating several downstream targets, mainly PKCs (18, 34). PLC was shown to be involved in keratin retraction, Dsg1 and Dsg3 reorganization and loss of adhesion *in vitro* (34). PLC inhibition prevented blister formation, ameliorated loss of desmosomes, desmosomal splitting, as well as keratin dissociation, but was not sufficient to fully rescue desmosome size in human skin *ex vivo* (17). Src directly phosphorylates e.g. PKP3, depleting it from the desmosomes (35), but also activates the epidermal growth factor receptor (EGFR) (36–38) in term activating a broad spectrum of further downstream targets (18). Src was shown to be involved in loss of adhesion *in vivo* (35, 39, 40), but was not effective in suppressing blister formation *ex vivo*, neither rescuing desmosome number nor size (17, 39). On the other hand, it was also reported, that Src activity is required for desmosome assembly to some degree (25). Erk1/2 downstream of MEK1/2, downstream of EGFR and thus Src and p38MAPK, was shown to impact Dsg1 and Dsg3 distribution and keratin retraction *in vitro* (17, 40). MEK1/2 inhibition prevented blister formation and partially rescued desmosome number but not reduction in desmosome size desmosomal splitting or keratin dissociation in human skin *ex vivo* (17, 40). Lastly, an increase in cAMP via adrenergic signaling modulators forskolin and rolipram was shown to ameliorate the fragmentation of Dsg3 staining and prevent loss of adhesion *in vitro*. Apremilast, a cAMP increasing drug already in clinical use (41), ameliorated fragmentation of Dsg3 staining, keratin retraction and prevented loss of adhesion *in vitro* and *in vivo*. It prevented blister formation but only rescued keratin insertion and desmosome splitting but not desmosome size or number in human skin *ex vivo* (42). However, only little is known about the impact of individual signaling pathways on the desmosome ultrastructure. The aim of this study was thus to systematically investigate the impact of several well-known pemphigus downstream signaling pathways on the desmosome ultrastructure *in vitro*. Using super resolution STED microscopy we specifically focused on Dp within the desmosomal plaque because it appears to be the most intriguing target for this kind of investigation. While Dp showed a complex reorganization (19), other plaque proteins like PG and PKP1-3 are mostly just depleted from the plaqueand to the cytoplasm or nucleus or degraded (43–51). The findings from our study might help to correlate signaling profiles to ultrastructural alterations and by extension to clinical outcomes. This could allow to develop new therapy options targeting specific steps of desmosome turn-over in the future.

## Results

### The composition of desmosomes in cultured cells is similar to that in intact epidermis

**Figure 1:**
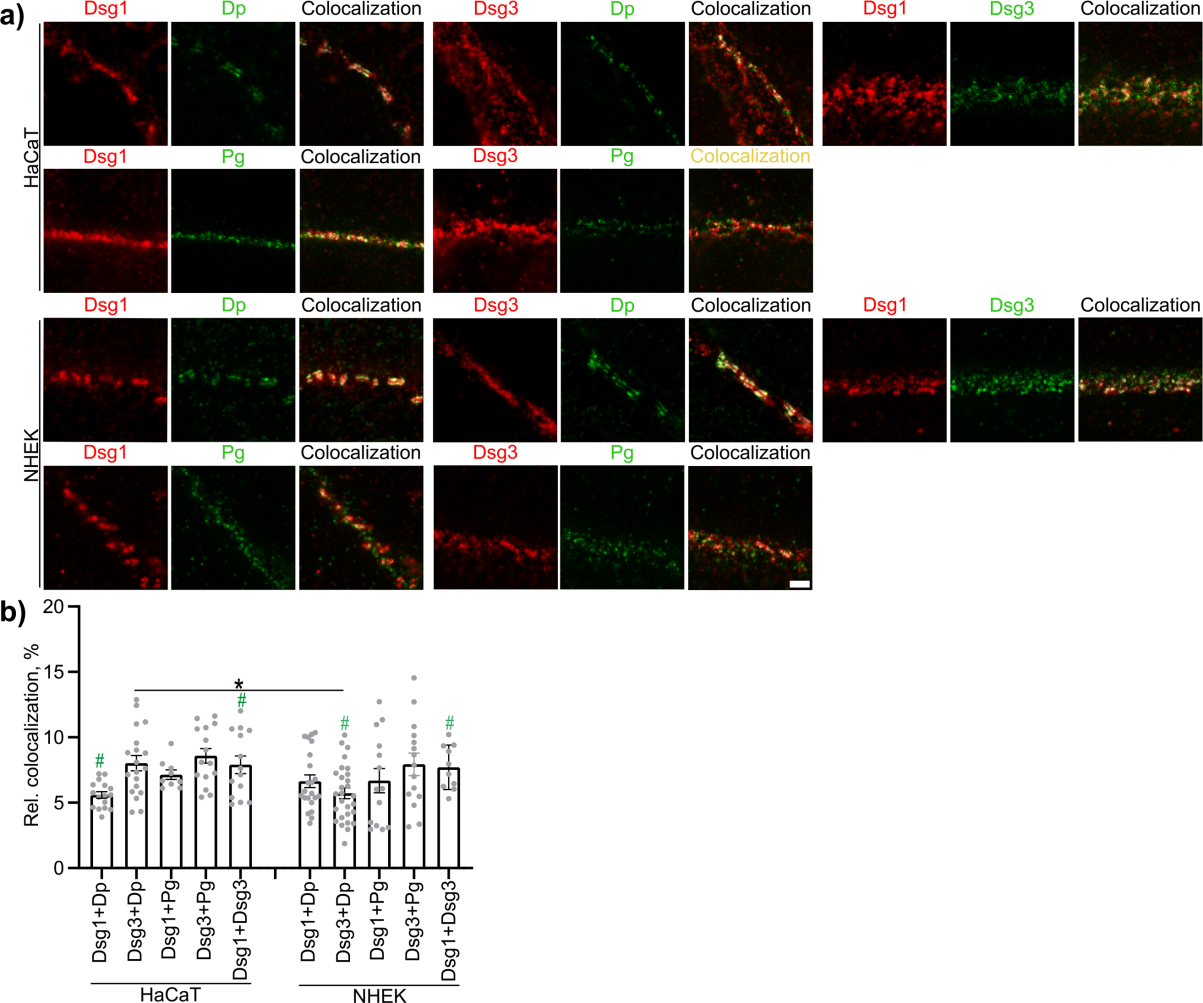
The composition of desmosomes in epidermal keratinocytes *in vitro*. **(A)** Representative STED-microscopy images (Scale bar: 500 nm). **(B)** Quantification results for relative area of colocalized pixels (N=4-5). * indicates statistically significant differences as indicated, # indicates statistical significant results as compared to epidermal keratinocytes in the spinous layer of human epidermis *ex vivo* as determined in a previous study (49), both in two-way-ANOVA with Sidaks post analysis for multiple comparisons p<0.05.

The first aim was to find out if the composition of desmosomes in cultured cells is comparable to that of epidermal desmosomes. The composition of desmosomes in the HaCaT cell line, as well as in primary NHEK cells, was mostly comparable to the composition of epidermal desmosomes located in the spinous layer (SL) as published previously (49). It was furthermore relatively similar between the two cell types, except for the colocalization of Dsg3 with Dp (**Figure 1**).

### Modulation of pemphigus signaling pathways protects against loss of adhesion in HaCaT cells *in vitro*

**Figure 2:**
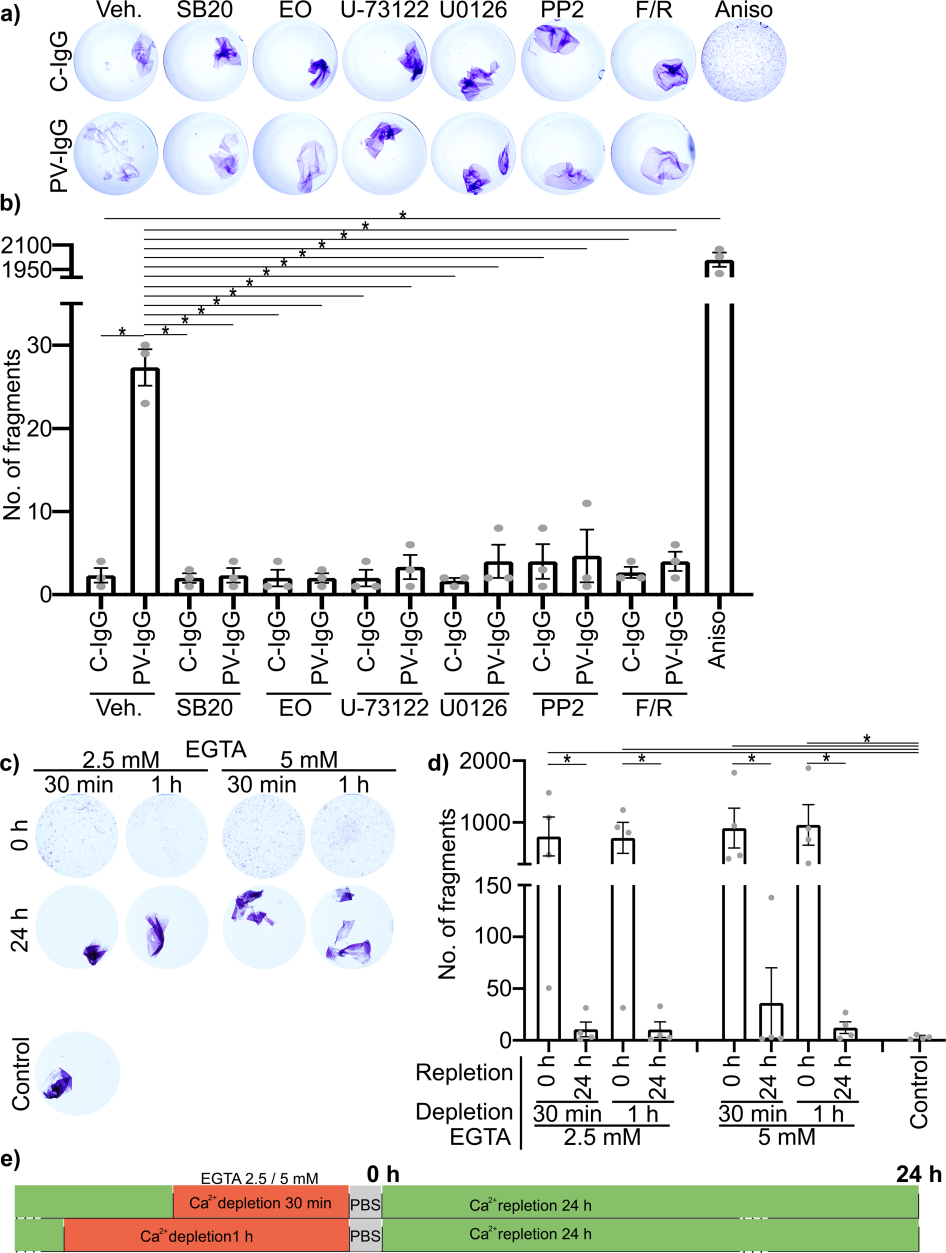
Impact of the used mediators and Ca^2+^-depletion on cell adhesion in HaCaT cells *in vitro*, as determined by dispase-based dissociation assays. **(A)** Representative Images of cells pretreated with mediators for 1 h followed by PV- or C-IgG for 24 h, stained for viability using MTT before sheering. **(B)** Quantification results for the number of fragments (N=3). **(C)** Representative Images of cells after depletion of Ca^2+^ with either 2.5 mM EGTA or 5 mM EGTA for 30 min or 1 h and with or without repletion of Ca^2+^ for 24 h. **(D)** Quantification results for the number of fragments (N=4). **(E)** Visual representation of the time course of Ca^2+^ depletion and repletion using EGTA. All experiments were performed using the human epidermal keratinocyte cell line: HaCaT. * indicates statistically significant differences as indicated in two-way-ANOVA with Sidaks post analysis for multiple comparisons p<0.05.

To confirm the protective effect on cell adhesion of each mediator to be used in this study the dispase-based dissociation assay was performed in HaCaT cells. These include inhibitors for the cell stress kinase p38MAPK, SB202190 (SB20) blocking the ATP-binding site (52) and EO1428 (EO) blocking the hydrophobic pocket, important for substrate binding and autophosphorylation (53); PP2 a potent inhibitor of Src-family kinases via a complex interaction, partially competing with ATP-binding (54); U-73122 inhibiting the PLC by blocking the binding of its upstream activating G-protein ligant (55). U0126 which inhibits the activation of MEK1/2 and increasing its dephosphorylation rate (52). Forskolin and activator of adenylate cyclase increasing activity by binding inside the catalytic pocket (56). Rolipram which inhibits degradation of cAMP by blocking Phosphodiesterase-4 activity by competitively binding to the catalytic domain, but also via a more complex allosteric binding mechanism (57). All mediators were effective in ameliorating the loss of adhesion induced by PV-IgG. None of the mediators showed any effect on baseline adhesion when applied with C-IgG. In contrast, activation of p38MAPK using Anisomycin (Aniso), induced activating phosphorylation of p38MAPK, and downstream ERK1/2, most likely via a general stress response (58–60), for 1 h induced severe fragmentation with about 2000 fragments and thus a factor of 100 more than PV-IgG (**Figures 2A, B**). It was confirmed that the fragmentation is not caused by cytotoxicity, using MTT vitality staining before sheering, producing a bright purple coloration of the monolayers under all conditions (**Figure 2A**). We compared cell adhesion after autoantibody treatment with cell adhesion after Ca^2+^-depletion. Complete disruption of cadherin-mediated adhesion by Ca^2+^-depletion for either 30 min or 1 h yielded around 2000 fragments with no significant difference between 2.5 mM or 5 mM EGTA. Repletion of Ca^2+^ for 24 h normalized adhesion for both concentrations of EGTA (**Figures 2C–E**).

### Modulation of pemphigus signaling restores PV-IgG-induced alterations in desmosome ultrastructure

**Figure 3:**
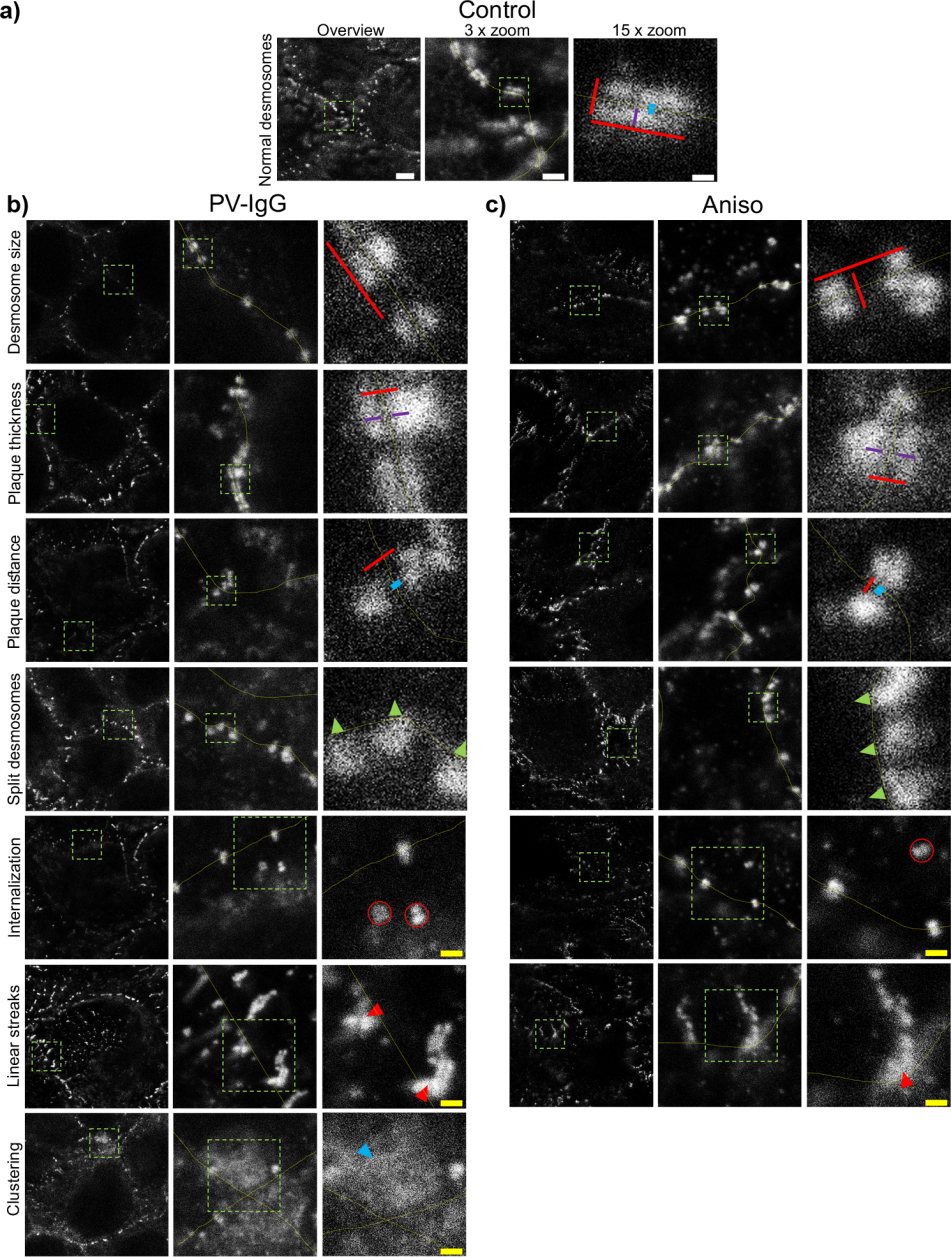
Qualitative summary of effects of PV-IgG or anisomycin treatment on the Dp plaque ultrastructure. Representative STED-microscopy images (Scale bars left to right 2 µm, 500 nm, 100 nm or yellow: 250 nm). **(A)** Control condition. **(B)** effects of PV-IgG treatment. **(C)** Effects of Anisomycin treatment. The lines indicate measurements of the example desmosome under control conditions. Red: length and width, purple: plaque thickness, blue: Plaque distance. Green arrows indicate missing halfs of split desmosomes, red arrows indicate linear streaks, blue arrows indicate areas where Dp staining is clustered and red circles indicate double Dp structures in the cytoplasm below the cell border. Green boxes indicate zoomed areas.

**Figure 4:**
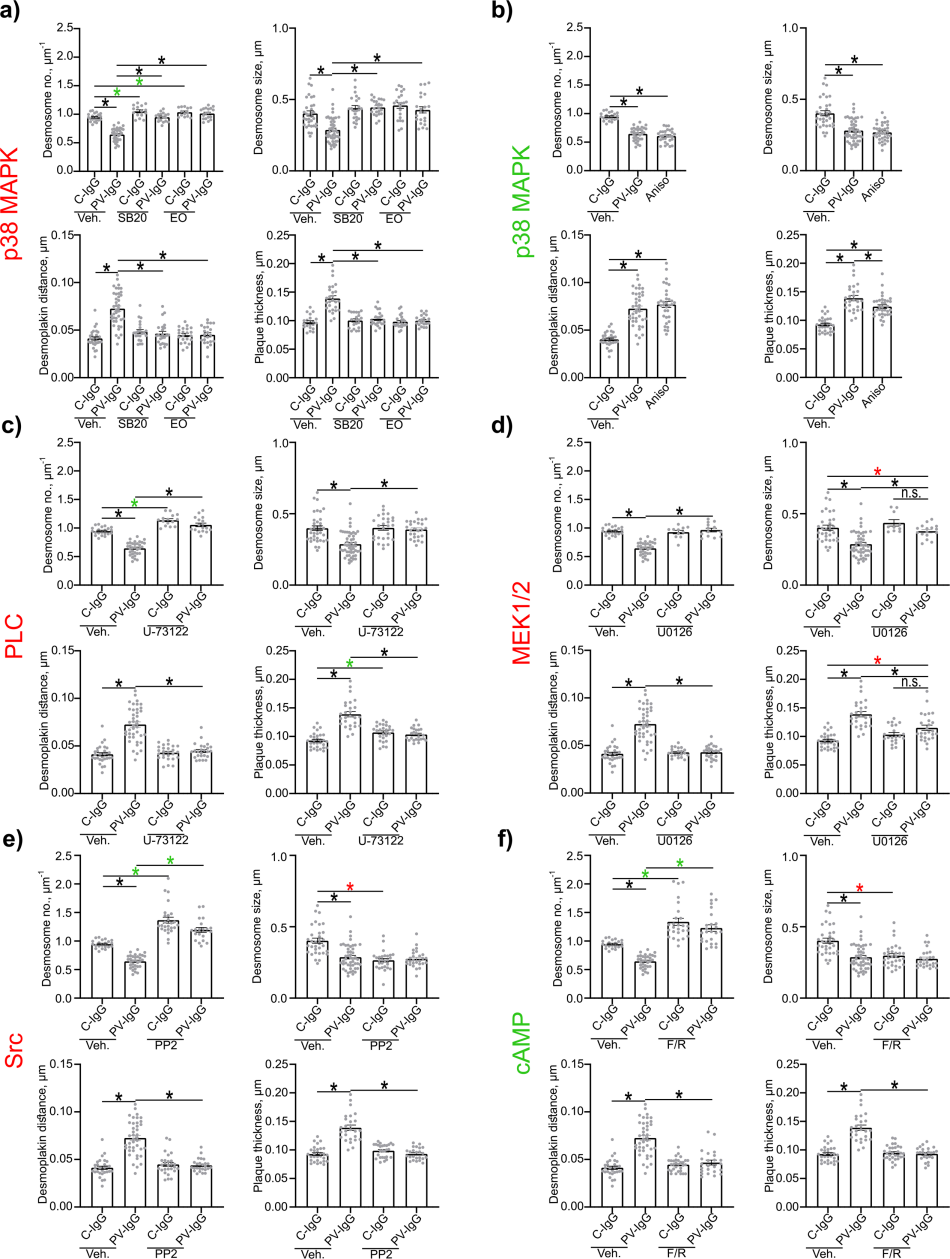
Quantification of effects of PV-IgG or anisomycin treatment on the Dp plaque ultrastructure. Quantification results for desmosome number, desmosome size, desmoplakin distance and plaque thickness (N=4-8). **(A)** inhibition of p38MAPK. **(B)** after activation of p38MAPK. **(C)** inhibition of PLC. **(D)** inhibition of MEK1/2. **(E)** inhibition of Src. **(F)** After induction of increased cAMP levels. * indicates statistically significant differences as indicated in two-way-ANOVA with Sidaks post analysis for multiple comparisons p<0.05.

**Figure 5:**
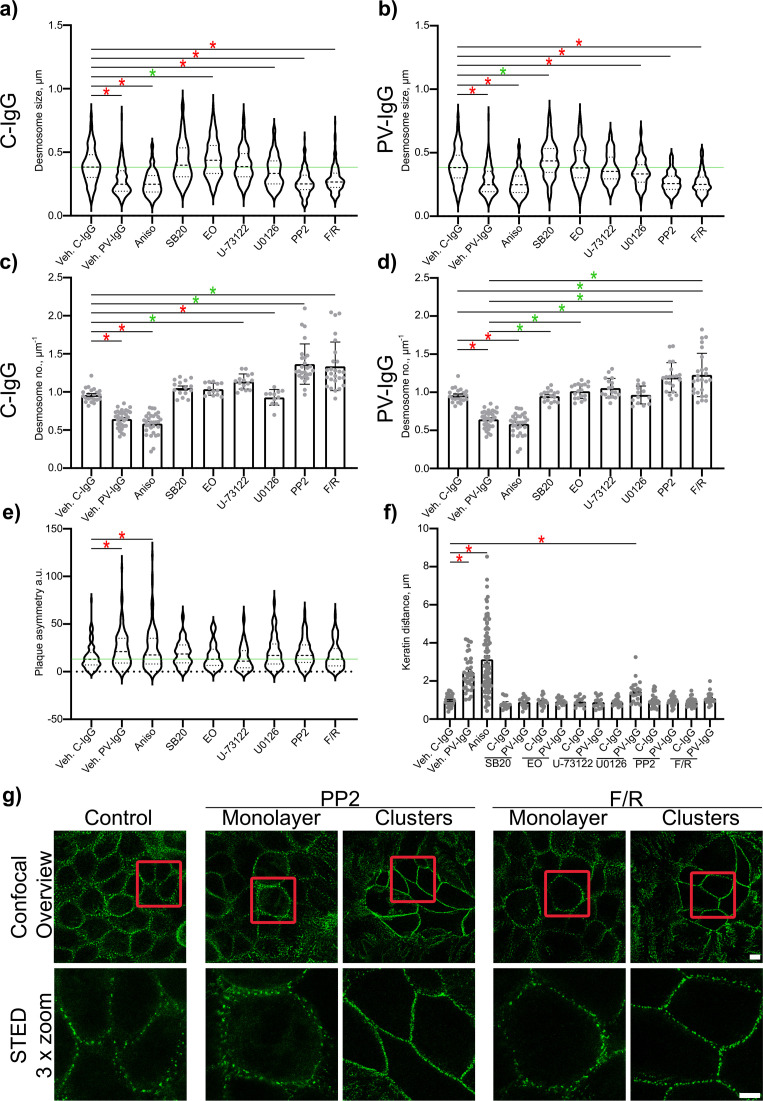
Quantitative summary of effects of PV-IgG or anisomycin treatment on the Dp plaque ultrastructure and evaluation of plaque symmetry and keratin retraction. **(A)** Quantification results for desmosome size for the subset after treatment with mediators and C-IgG compared to vehicle control for c-IgG, PV-IgG and anisomycin (N=4-8). **(B)** Quantification results for desmosome size for the subset after treatment with mediators and PV-IgG compared to the vehicle control for c-IgG, PV-IgG and anisomycin (controls are the same as for a) N=4-8). **(C)** Quantification results for desmosome number for the subset after treatment with mediators and C-IgG compared to vehicle controls for C-IgG, PV-IgG and anisomycin (N=4-8). **(D)** Quantification results for desmosome number for the subset after treatment with mediators and PV-IgG compared to the vehicle control for c-IgG, PV-IgG and anisomycin (controls are the same as for C) N=4-8). **(E)** Quantification results for plaque asymmetry after treatment with mediators and C-IgG compared to PV-IgG, anisomycin and control conditions (N=4-8). **(F)** Quantification results for keratin distance (keratin retraction) after treatment with mediators and C-IgG or PV-IgG (N=4-8). **(G)** Representative STED-microscopy images for delaminated cells showing more desmosomes along the cell borders after PP2 or F/R treatment, compared to cells in the monolayer showing still more desmosomes than under control conditions (Red boxes indicate zoomed in area. Scale bar top: 2 µm; bottom: 10 µm). * indicates statistically significant differences as indicated in two-way-ANOVA with Sidaks post analysis for multiple comparisons p<0.05.

Evaluation of Dp plaque ultrastructure using STED microscopy revealed that PV-IgG-induced several alterations compared to desmosomes under control conditions (**Figures 3A, B**). Both, the number of desmosomes per µm of cell border length and desmosome size were significantly decreased (**Figures 3A**, **4**, **5A–D**). The plaque thickness was increased (**Figures 3A**, **4**), and some desmosomes showed asymmetry between plaques of neighboring cells (**Figure 5E**) indicating plaque disorganization. The distance between the desmosomal plaque sub-cells was increased (**Figures 3A**, **4**). In regions where the cell borders had already drifted apart, half/split desmosomes were observed (**Figure 3A**, green arrowheads). At some cell borders either single desmosome-like structures in the cytoplasm or linear streaks with desmosome-like structures arranged in lines were present inside the cytoplasm, indicating desmosome remodeling (**Figure 3A**, red arrowheads/circles). Lastly, in some areas, clusters of Dp staining were present (**Figure 3A**, blue arrowhead). After activation of p38MAPK with anisomycin, similar effects were observed for all parameters of desmosome ultrastructure. However, clustering of Dp staining was absent (**Figures 3C**, **4B**, **5A–D**).

The inhibition of p38MAPK with either SB20190 (SB20) or EO1485 (EO) but also inhibition of PLC with U-73122 ameliorated all PV-IgG-induced ultrastructural alterations and even slightly increased the number of desmosomes (**Figures 4A, C**, **5C, D**). Inhibition of MEK1/2, which is upstream of Erk1/2, with U0126, ameliorated the loss of number of desmosomes but only partially restored desmosome size. Increased plaque distance was rescued but increase of plaque thickness was only partially reduced (**Figures 4D**, **5A–D**). Both, inhibition of Src via PP2 and increase of cAMP levels using F/R strongly increased the number of desmosomes and desmosome number was not decreased by PV-IgG. However, desmosome size was decreased to a similar degree as after incubation with PV-IgG. Both plaque distance and thickness were protected after addition of PP2 or F/R (**Figures 4E, F**, **5A, B, G**). Under both conditions, clusters of delaminated cells, with even more desmosomes than the surrounding cells were present (**Figure 5G**). The desmosomal plaque asymmetry induced by PV-IgG and anisomycin was abolished by all mediators (**Figure 5E**).

**Figure 6:**
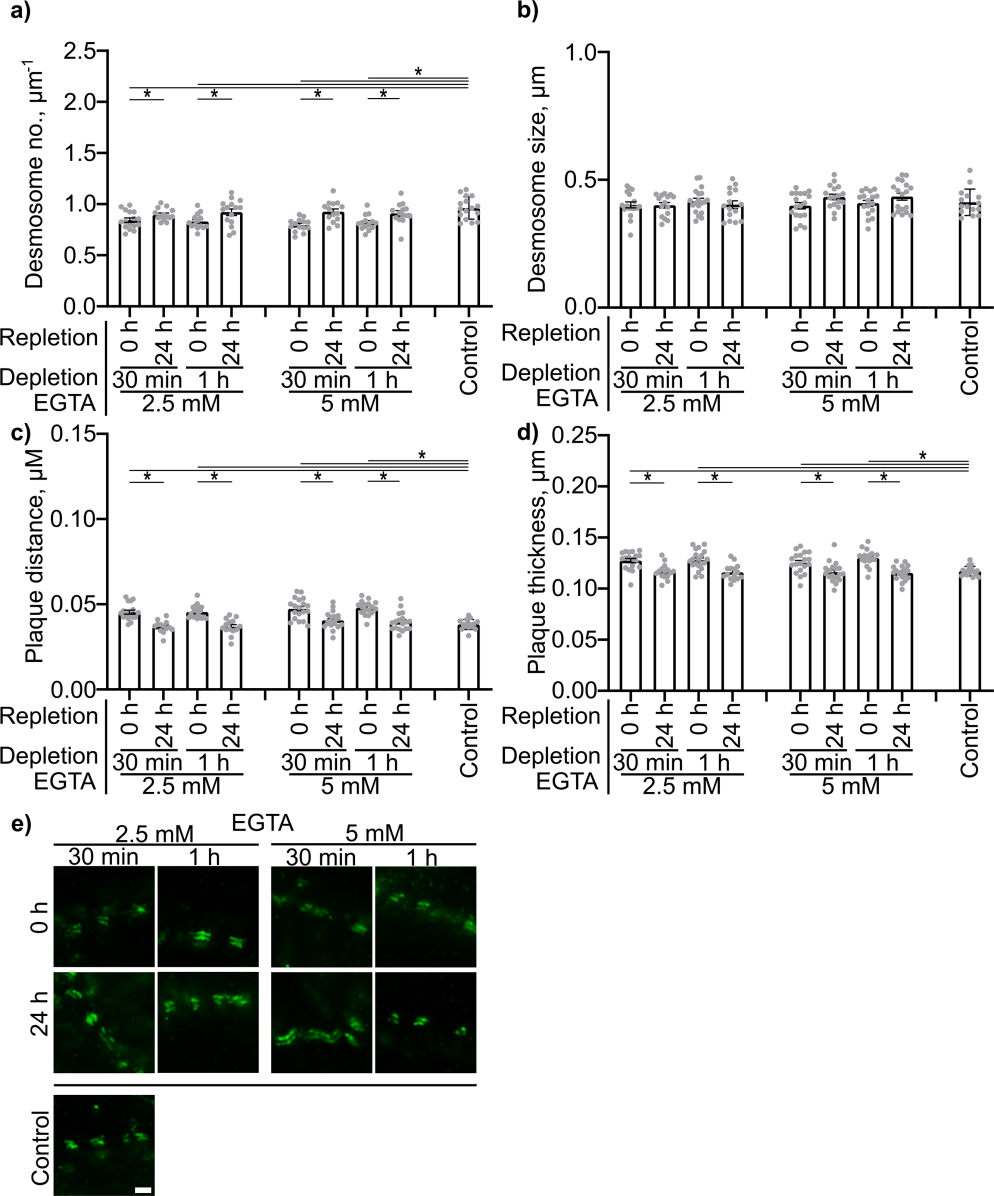
Quantification of effects of Ca^2+^ depletion and repletion on the Dp plaque ultrastructure. Quantification results for **(A)** desmosome number. **(B)** desmosome size. **(C)** desmoplakin distance. **(D)** plaque thickness (N=4). **(E)** Representative STED-microscopy images showing alterations in the Dp plaque ulstrastructure (Scale bar: 500 nm). * indicates statistically significant differences as indicated in two-way-ANOVA with Sidaks post analysis for multiple comparisons p<0.05.

PV-IgG- and p38MAPK activation-induced alterations, including decreased desmosome number, increased plaque distance and thickness and loss of adhesion, were comparable to features observed for newly formed immature desmosomes, after depletion of Ca^2+^ for 30 min or 1 h with 2.5 or 5 mM EGTA as described above. Repletion for 24 h completely restored the normal ultrastructure of desmosomes (**Figure 6**).

### Signaling pathway modulation prevents PV-IgG-induced alterations in the distribution of desmosomal proteins

**Figure 7:**
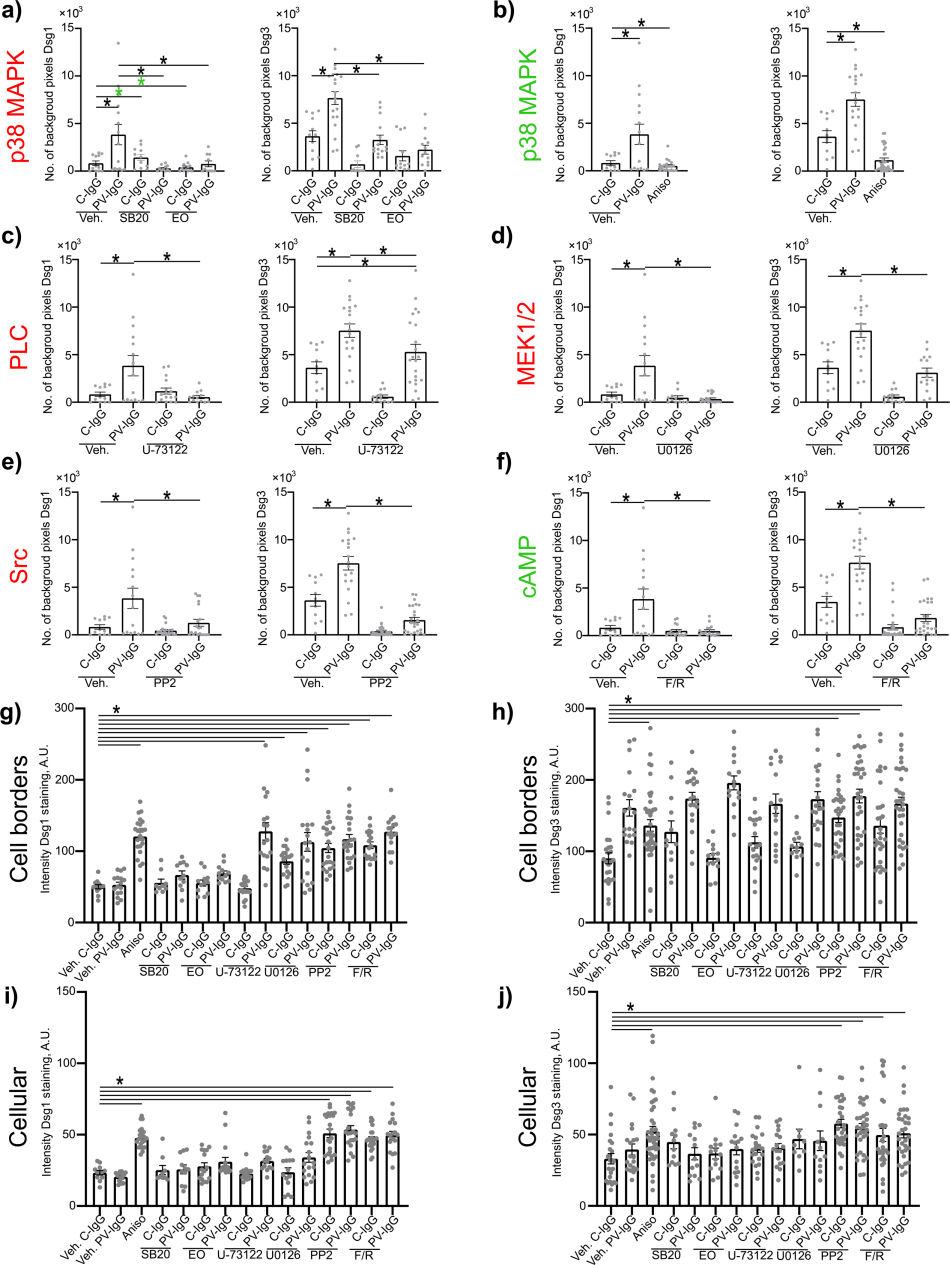
Quantification of effects of PV-IgG or anisomycin on Dsg1 and Dsg3 staining distribution and intensity. Quantification results for fragmentation of Dsg1 and Dsg3 (N=3-5). **(A)** Inhibition of p38MAPK. **(B)** activation of p38MAPK **(C)** inhibition of PLC **(D)** inhibition of MEK1/2 **(E)** inhibition of Src **(F)** After induction of increased cAMP levels. **(G)** Quantification results for Dsg1 staining intensity along the cell borders after treatment with mediators and C-IgG or PV-IgG (N=3-5). **(G)** Quantification results for Dsg1 staining intensity along the cell borders after treatment with mediators and C-IgG or PV-IgG (N=3-5). **(H)** Quantification results for Dsg3 staining intensity along the cell borders after treatment with mediators and C-IgG or PV-IgG (N=4-5). **(I)** Quantification results for Dsg1 staining intensity in the cytoplasm after treatment with mediators and C-IgG or PV-IgG (N=3-5). **(J)** Quantification results Dsg3 staining intensity in the cytoplasm after treatment with mediators and C-IgG or PV-IgG (N=4-5). * indicates statistically significant differences as indicated in two-way-ANOVA with Sidaks post analysis for multiple comparisons p<0.05.

**Figure 8:**
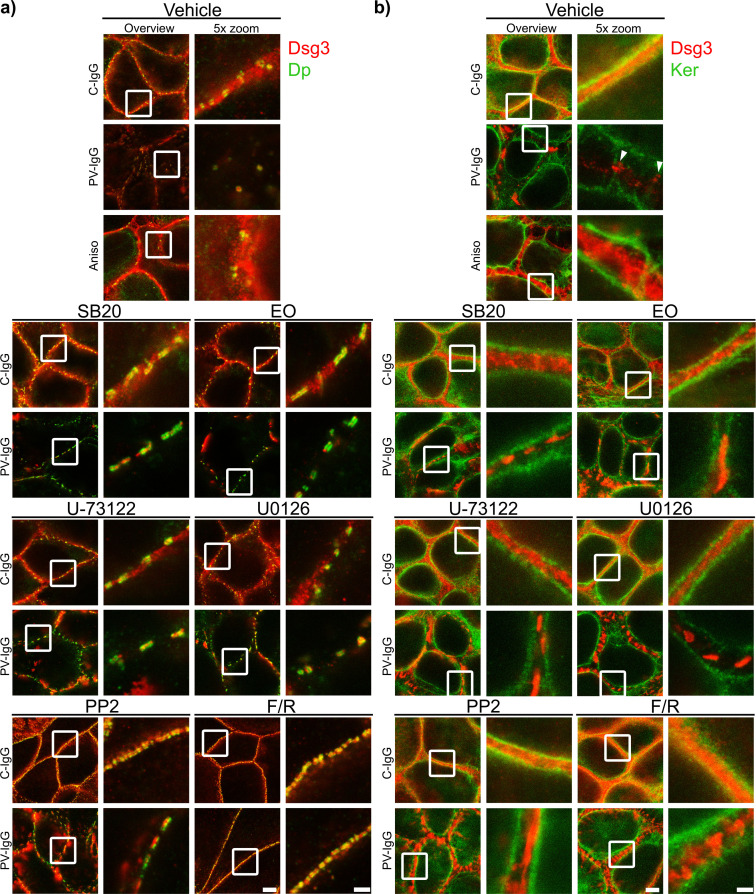
Qualitative display of the impact of mediators on the distribution and association of Dsg3 with Dp and keratin filaments. Representative STED-microscopy images after 1 h pretreatment with mediators followed by 24 h of treatment with C-IgG or PV-IgG for: **(A)** Costaining of Dsg3 and Dp. **(B)** Costaining of Dsg3 and pan-cytokeratin (Ker). (White arrows indicate Dsg3 associated with keratin filaments. White boxes indicate zoomed in area. Scale bar left to right: 2 µm, 500 nm).

**Figure 9:**
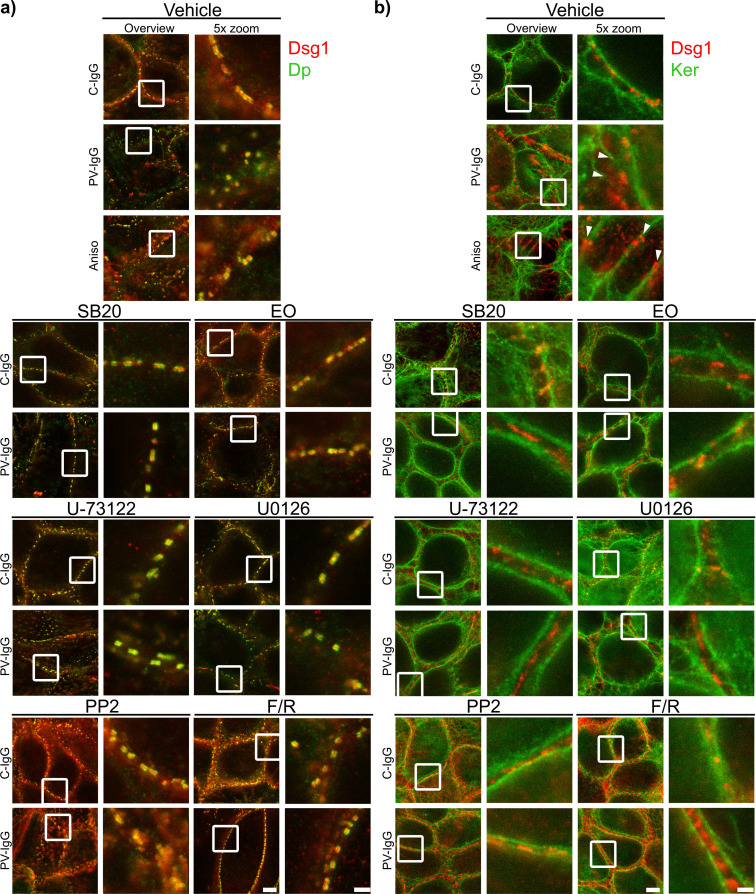
Qualitative display of the impact of mediators on the distribution and association of Dsg1 with Dp and keratin filaments. Representative STED-microscopy images after 1 h pretreatment with mediators followed by 24 h of treatment with C-IgG or PV-IgG for: **(A)** Costaining of Dsg1 and Dp. **(B)** Costaining of Dsg1 and pan-cytokeratin (Ker). (White arrows indicate Dsg1 associated with keratin filaments. White boxes indicate zoomed in area. Scale bar left to right: 2 µm, 500 nm).

Both PV-IgG and anisomycin induced retraction of keratin filaments from cell borders toward the nucleus (**Figure 5F**). Keratin retraction induced by PV-IgG was prevented by all mediators except U0126, which only reduced it. Alterations induced by PV-IgG and anisomycin furthermore included fragmented staining of Dsg1 and Dsg3 (**Figures 7**–**9**), with mostly desmosomal Dsg staining remaining as visible by the keratin insertion (**Figures 8B**, **9B**, white arrowheads). Anisomycin as well as U0126, PP2 and F/R increased Dsg1 staining at the membrane and anisomycin (**Figures 7G, H**), PP2 and F/R also enhanced Dsg3 staining intensity both at the membrane and in the cytoplasm (**Figures 7G–J**). The fragmentation of Dsg1 and Dsg3 staining was normalized by all mediators, only exception was U-73122 which reduced Dsg3 fragmentation but did not normalize it (**Figures 7A, C, F**, **8**, **9**). U-73122 did also not normalize Dsg3 staining intensity (**Figure 7**). p38MAPK activation by anisomycin caused an increased Dsg intensity both at cell borders and in the cytoplasm (**Figures 7G–J**).

## Discussion

Our results suggest that PV-IgG alters desmosome ultrastructure to a state similar to immature desmosomes. This seems to be mediated in part via p38MAPK but also other signaling pathways including PLC, MEK1/2 (Erk1/2), Src and cAMP. While many of the signaling molecules showed similar effects, there were some interesting differences such as only partial rescue of plaque thickness by inhibition of MEK1/2 or more but smaller desmosomes upon inhibition of Src or increase of cAMP. These differences might be important to identify distinct steps of pemphigus pathogenesis and to establish specific therapy approaches targeting these in the future.

### Desmosomes in HaCaTs are most similar to those at the epidermal basal layer-spinous layer interface

First important step was to check whether the findings on desmosome ultrastructure in cultured cells are comparable to the situation in keratinocytes of human epidermis. For this we verified that the desmosomes in both the HaCaT cell line and primary NHEK cells are comparable to desmosomes found in epidermis of body doners and control sample from non-pemphigus patients *ex vivo*. Overall, desmosomes in both cell types investigated represented epidermal desmosomes found in the SL of the epidermis quite well. SL desmosomes showed the overall highest colocalization for Dsg3+Dp, Dsg1+Dp and Pg+Dp compared to the other layers, and very similar to the cells, while Dsg1+Pg was similar across all layers. The only difference was that both HaCaTs and NHEKs were more homogeneous with respect to Dsg1/Dsg3 composition whereas in the skin, desmosomes mostly contain either Dsg1 or Dsg3, with the highest degree of colocalization of the to in the basal layer (BL) (49). The only difference between HaCaTs and NHEKs was the colocalization of Dsg1 and Dsg3 with Dp. NHEKS showed a bit less colocalization of Dsg3 with Dp, while HaCaTs showed a bit lower colocalization of Dsg1 with Dp. Both were respectively significantly different to the SL composition. Since the higher colocalization of Dsg3 and lower colocalization of Dsg1 with Dp is more similar to the BL, HaCaTs apparently represent desmosomes at the basal-suprabasal interface of the epidermis. Because autoantibodies in PV induce splitting at this interface, all further experiments on regulation of the desmosome ultrastructure were performed with HaCaT cells (61, 62).

### Pemphigus-IgG-induced signaling reverts desmosomes to an immature state

It was previously shown that the composition of desmosomes was not drastically altered regarding Dsg1, Dsg3, Pg and Dp in PV patient skin, compared to controls, neither in lesional, nor in non-lesional regions. However, the number of desmosomes per membrane length was significantly reduced, causing reduced adhesion and blister formation (19, 49). Electron microscopy and recently also STED and dSTORM microscopy further revealed PV-IgG-induced alterations of the desmosomal plaque. These also include reduced desmosome number (19, 63, 64) but furthermore reduced plaque size (19, 20, 33, 63–66), increased inter plaque distance (19, 20), increased plaque thickness and/or disorganization (4, 19), formation of split desmosomes (33, 63, 64) as well as altered keratin organization and insertion (4, 19, 63). These alterations can be concluded to be both a good indicator for, as well as a cause for the reduced desmosome adhesion. Using STED microscopy, we demonstrated that most alterations of the desmosome ultrastructure were comparable after incubation with PV-IgG or following activation of p38MAPK via anisomycin conforming a previous study (19). We found that the plaques of affected desmosomes are often asymmetrical. In contrast, clustering of desmosomal components was observed only after treatment with PV-IgG, identifying this effect to be p38MAPK-independent. Most of these alterations, including decreased desmosome number, increased interplaque distance and thickness as well as loss of adhesion closely resemble features found in newly assembled immature desmosomes as observed after Ca^2+^ depletion, also confirming previous reports (20, 67, 68). This strongly suggests that PV-IgG-induced signaling stimulates desmosome assembly pathways and reverts the desmosome ultrastructure back to a state similar to that of immature desmosomes or desmosome precursors. This also fits to previous reports about the importance of several PV-IgG-induced signaling pathways responsible for desmosome assembly and maturation, including PKC (69–72), RhoA (39, 73–75), Src (25, 35, 39), and Rap1 (76).

### Signaling pathways play distinct roles in the regulation of the desmosomal Dp plaque ultrastructure

Next we wanted to investigate in more detail how modulation of different signaling pathways involved in pemphigus pathogenesis affected the desmosome ultrastructure. It is well known that p38MAPK is involved in pemphigus pathogenesis (18). Similar to previous reports, our results demonstrate that p38MAPK is directly involved in loss of adhesion (31, 32). We also confirm the involvement of p38MAPK in keratin retraction (28–30, 65) and alterations of Dsg3 distribution (21, 26, 27). Additionally, we observed that p38MAKP affected the distribution of Dsg1 in a similar fashion. p38MAKP inhibition ameliorated all alterations to the desmosomal plaque ultrastructure, which fits to previous findings using electron microscopy, reporting reduced number of desmosomes, decreased desmosome size, interdesmosomal widening, split desmosomes and altered keratin insertion (65). The slight increase in the number of desmosomes further indicates that also under basal conditions p38MAPK negatively regulates desmosome assembly.

We further investigated the role of PLC. Similarly to p38MAPK, PLC seems to regulate all parameters of desmosome ultrastructure as well as keratin filament organization. This mostly fits to previous reports showing that inhibition of PLC and its downstream targets was protective against keratin retraction and loss of adhesion (34, 63). For Desmosome number, a previous study reported a similar trend in human skin *ex vivo.* However, with not significant difference, possibly due to the weak effect and high interindividual variability in the investigated *ex vivo* skin (17, 63). This may also explain why desmosome length was not rescued. Interestingly, inhibition of PLC fully prevented fragmentation of Dsg1 staining whereas effects on Dsg3 staining were less pronounced. That might indicate that PLC impacts extradesmosomal Dsg1 more than extradesmosomal Dsg3. However, the at least partial rescue of both confirms previous findings (34).

The data are in line with the hypothesis that both p38MAPK and PLC are relatively far upstream in the signaling mechanisms involved in desmosome dysregulation in PV (18). In contrast, inhibition of MEK1/2, downstream of Src, p38MAPK and EGFR (18), improved but did not fully rescue desmosome size, plaque thickness or keratin retraction, which may indicate that MEK1/2 is further downstream in the signaling mechanisms after autoantibody binding and may regulate desmosome turn-over at specific steps only. While loss of number of desmosomes and plague distance were rescued, the desmosome size and plaque thickness were only partially normalized. This fits well with results from a previous electron microscopy study showing that neither desmosome size, desmosomal splitting, nor keratin dissociation were rescued in human skin *ex vivo* (17, 66).

Both, inhibition of Src and increase of cAMP levels showed very distinct effects compared to the signaling pathways outlined above. Both signaling pathways rescued most desmosomal parameters and keratin insertion which is similar to inhibition of p38MAPK and PLC. However, under control conditions Src inhibition and cAMP increase resulted in a higher number of smaller desmosomes and increased staining of Dsg1 and Dsg3 along the membrane and in the cytoplasm. A possible interpretation would be that desmosome assembly was accelerated which prevented desmosomes to fully mature. The increased Dsg3 staining could result from an increased amount of Dsg3 containing desmosomal precursors. This fits well with previous reports on the protective role of Src and cortactin described to regulate the integration of desmosome precursors into desmosomes (25, 35, 39). In contrast to that, Src was not protective in preventing blister formation in human skin *ex vivo* and did not resuce desmosome number, size nor inderdesmosomal widening, but was effective in preventing blister formation in neonatal mouse skin *in vivo* (17, 39). This could possibly indicating, that for Src inhibition to be protective more dynamic reorganization needs to be in process. Src for example acts via the epidermal EGFR (36–38), which might show a different regulation in differentiated human skin than in cultured cells. It was furthermore reported, that Src was no longer active in skin after 24 h and Src inhibition was only protective during the timeframe, while it was active (39). This partially confirms the need for active reorganization processes to be going on for Src inhibition to be protective. For cAMP, these observations also conform to a previous report demonstrating its protective effects in mice *in vivo* and keratinocytes *in vitro*, for which keratin insertion were rescued, while desmosome size was not rescued, but in contrast to *in vitro* findings also not reported to be reduced (42). That the size of desmosomes was not reduced might, similarly as for Src, indicate, that differentiated adult human skin shows less dynamic desmosome reorganization than cells *in vitro*. Another study demonstrated that Rap1 downstream of cAMP affects plaque formation and thus desmosome assembly via Pkp3 (76), which also fits well to this hypothesis.

### Conclusion: Signaling molecules rescuing desmosome ultrastructure may become targets for new therapy approaches in pemphigus

From our data, it can be concluded that modulation of the pemphigus signaling network downstream of autoantibody binding at different points may allow to rescue desmosome adhesion as has been shown in many studies before (18). However, in the recent study we observed that the effects of signaling pathways on the desmosomes ultrastructure may be very different which may indicate the role of these signaling mechanisms in the different steps of desmosome turn-over and the hierarchical position of the respective molecules in the pemphigus signaling network. Molecules more upstream in the corresponding signaling chain and thus activated earlier, upon autoantibody binding, such as p38MAPK and PLC affect all parameters of desmosome ultrastructure whereas others, including MEK1/2, may be more downstream of autoantibody binding or involved only in distinct steps of desmosome turn-over. For example, MEK1/2 appears to stabilize mature desmosomes but is less important for the regulation of cytoskeletal anchorage of desmosomes. In contrast, we conclude that both Src and cAMP most likely are involved in the regulation of desmosome assembly.

These new insights may be important in the future to establish new treatment options in pemphigus. At this stage, treatment is limited to immunosuppression and B cell depletion which is associated with side effects and considerable relapse rates and new experimental strategies also focus on the reduction of autoantibody levels (77). Therefore, new treatment options are required (78) and we proposed to stabilize keratinocyte adhesion by promoting desmosome adhesion for example via apremilast to enhance cAMP levels or erlotinib to inhibit EGFR (19, 42). Since in this context, Src families associated with EGFR activation were found to be activated by autoantibodies, our new data indicate that therapy options to enhance desmosome assembly may be effective to treat pemphigus in the future.

## Materials and methods

### Cell culture

The human epidermal keratinocyte cell line HaCaT was cultivated in a humidified, 5% CO_2_ atmosphere at 37°C in Dulbecco’s Modified Eagle Medium (DMEM) (Life Technologies, CA) with 10% FCS (Merk, DE) and 50 U/ml penicillin and 50 µg/ml streptomycin (both AppliChem, DE). Primary normal human epidermal keratinocytes (NHEK) in passages 2-6 in CnT-Prime Basal Medium 1 at low calcium (0.06 mM) until confluency and 1.8 mM calcium for differentiation for 24 h after reaching confluency. Passaging and seeding was performed, using trypsin-EDTA solution (Merck, DE). For Ca^2+^ depletion the cells were treated with EGTA 2.5 or 5 mM for 30 min or 1 h. Repletion was performed by washing with PBS 1x and exchanging to new medium.

### Mediators and staining antibodies

Cells were pre-incubated with PP2 for 2 h or all other mediators for 1 h before IgG treatment for 24 h. Anisomycin was incubated for 1 h before fixing the cells. For concentration of mediators refer to **Table 1**, for staining antibodies refer to **Table 2**.

**Table 1:** Mediators.

Mediator	Target	Function	Concentration		Cat. No.	Supplier
–––	DMSO/PBS	Vehicle	1:1000		276855	Sigma Aldich USA
SB202190 (SB20)	p38MAPK	Inhibitor	60	µM	8158	Cell signaling, USA
EO1428 (EO)	p38MAPK	Inhibitor	40	nM	2908	Torcis Bio-Techne, DE
Anisomycin	p38MAPK	Activator	60	µM	10522	Sigma Aldich USA
U0126	MEK1/2	Inhibitor	5	µM	9903	Cell signaling, USA
PP2	Src	Inhibitor	10	µM	529573	Calbiochem, DE
U-73122	PLC	Inhibitor	4	µM	sc-3574	Santa Cruz, USA
Forskolin	Adenylate cyclase	Activator	10	µM	F3917	Sigma Aldich USA
Rolipram	Phosphodiesterase-4	Inhibitor	5	µM	R6520	Sigma Aldich USA
EGTA	Ca^2+^	Chelator	2.5/5	mM	0732	VWR, DE

**Table 2:** Staining antibodies.

Antibody	Species	Cat. No.	Supplier	Ratio
anti-Dsg1-pAb	Rabbit	A9812	Abclonal, CN	1:200
anti-Dsg3-pAb	Rabbit	62720-120	Biozol, DE	1:200
Anti-cytokeratin-pan-mAb	Mouse	C2931	Sigma Aldich USA	1:200
anti-Dp -pAb	Mouse	A13299	Abcam, GB	1:200

### Pemphigus sera and IgG

PV-IgG with ELISA scores for anti-Dsg1-IgG: 1207.00 U/ml and anti-Dsg3: 3906.00 U/ml was obtained as immunoapharesis purified material from the Lübeck Institute of Experimental Dermatology. Protein A agarose beads (Thermo Fisher Scientific, USA) were used for affinity purification to obtain IgG fractions from sera of healthy volunteers as control-IgG (C-IgG), as described previously (24). IgGs were used 1:50 in cell culture experiments and about 50 µl/cm² of skin ex vivo.

### Dispase-based dissociation assay

Confluent HaCaT monolayers were washed with Hank´s buffered saline solution (HBSS). 150 µl Dispase II, 2.4 U/ml (Sigma Aldrich, USA) in HBSS was added for 10 min at 37°C, 95% humidity. After monolayer detachment the dispase reaction was stopped by diluting with 200 µl HBSS. The monolayers were stained with MTT (VWR, DE) to confirm viability and produce a better contrast. An electrical pipette was used to induce defined shear stress. Fragment numbers were counted using images taken, using a binocular microscope (Leica, DE) and an EOS 600D camera (Canon, JP).

### Human skin samples

Skin biopsies from body donors deceased for less than 24 h were used. Each epidermis piece ~6 cm² was excised from the shoulder region and divided into ~1 cm² pieces. The skin was cultivated in floating culture, for 24 h after the second injection, in 6-well-plates in 4 ml medium under the same conditions as the keratinocytes. After 24 h, the samples were cut into shape about ~0.4 cm² embedded in tissue freezing medium (Leica, DE). 7 μm sections were produced, using a CryoStar™ NX70 Kryostat (Thermo Fisher, USA).

### STED (Stimulated emission depletion) microscopy

Treated cells grown on high performance glass cover slips or skin slices were fixed using -20°C ethanol for 30 min followed by -20°C acetone for 3 min. Primary antibodies were incubated for 3 h at RT and secondary goat-anti-mouse or -rabbit antibodies coupled to STAR-RED or Alexa 594 fluorophores (Abberior GmbH, Göttingen, DE) for 1 h. DAPI (Roche, DE) was added for the last 15 min of secondary antibody incubation. Using Prolong™ Diamond Antifade Mountant, cover slips were mounted to glass slides (Thermo Fisher Scientific, DE). An Abberior 3D Stimulated emission depletion (STED) confocal microscope with IMMOIL-F30CC (Olympus, JP) was used to image the samples. Star Red was excited at 638 nm and Alexa594 at 594 nm, using a pulsed diode lasers (PDL 594, Abberior Instruments; PiL063X, Advanced Laser Diode Systems). Fluorophores were depleted using a pulsed fiber laser (PFL-P-30-775B1R, MPB Communications) at 775 nm. Emission was detected with an avalanche photodiode detector at 605-625 nm for Star Red and 650-720 nm for Alexa 594.

### Image processing and statistical analysis

Image processing was performed with Photoshop CS5. Image J was used to quantify fluorescence staining (IF). Each data point represents an average per STED image. All images were evaluated, using Fiji Image J software v 2.9.0. Data were analyzed using either two-way-ANOVA followed by Sidak-*post-hoc*-test for multiple comparison or if applicable one-way-ANOVA followed by Dunnett-*post-hoc*-test for multiple comparison, using GraphPad Prism v8.4.3 (GraphPad Software, USA). Significance was assumed for p ≤ 0.05. Data are shown as mean ± standard error of the mean. Each N represents one independent experiment, each n a technical replicate.

## Data Availability

The raw data supporting the conclusions of this article will be made available by the authors, without undue reservation.
